# Robust methods for population stratification in genome wide association studies

**DOI:** 10.1186/1471-2105-14-132

**Published:** 2013-04-19

**Authors:** Li Liu, Donghui Zhang, Hong Liu, Christopher Arendt

**Affiliations:** 1 Department of Biostatistics and Programming, Mail Stop 55C-305A, 55 Corporate Drive, Sanofi, Bridgewater, NJ, 08807, USA; 2 Bio-Innovation Group of Sanofi Biotherapeutics, 38 Sidney Street, Sanofi, Cambridge, MA, 02142, USA

**Keywords:** Population structure, Population stratification, Robust principal component analysis, Resampling by half means, Outlier detection, GWA studies

## Abstract

**Background:**

Genome-wide association studies can provide novel insights into diseases of interest, as well as to the responsiveness of an individual to specific treatments. In such studies, it is very important to correct for population stratification, which refers to allele frequency differences between cases and controls due to systematic ancestry differences. Population stratification can cause spurious associations if not adjusted properly. The principal component analysis (PCA) method has been relied upon as a highly useful methodology to adjust for population stratification in these types of large-scale studies. Recently, the linear mixed model (LMM) has also been proposed to account for family structure or cryptic relatedness. However, neither of these approaches may be optimal in properly correcting for sample structures in the presence of subject outliers.

**Results:**

We propose to use robust PCA combined with k-medoids clustering to deal with population stratification. This approach can adjust for population stratification for both continuous and discrete populations with subject outliers, and it can be considered as an extension of the PCA method and the multidimensional scaling (MDS) method. Through simulation studies, we compare the performance of our proposed methods with several widely used stratification methods, including PCA and MDS. We show that subject outliers can greatly influence the analysis results from several existing methods, while our proposed robust population stratification methods perform very well for both discrete and admixed populations with subject outliers. We illustrate the new method using data from a rheumatoid arthritis study.

**Conclusions:**

We demonstrate that subject outliers can greatly influence the analysis result in GWA studies, and propose robust methods for dealing with population stratification that outperform existing population stratification methods in the presence of subject outliers.

## Background

In genome-wide association (GWA) studies, hundreds of thousands of single-nucleotide polymorphisms (SNPs) are assayed using high-throughput genotyping technologies and are tested for their associations with clinical outcomes of interest. The new genetic associations identified by these studies can be used to improve the detection, treatment and prevention of certain diseases, particularly when used in conjunction with other clinical biomarkers. For example, individuals may be identified who are more likely to respond to a specific treatment while experiencing fewer side effects. Eventually, each patient may be able to receive his/her personalized treatment instead of a one-size-fits-all treatment.

To date, the most frequently used GWA study design has been the case-control design, in which allele frequencies in patients with the disease (cases) are compared to those without the disease (controls) among unrelated individuals, or allele frequencies in patients who responded to the treatment are compared to those who did not respond to the treatment. Compared to family-based designs, the case-control studies are usually less expensive and easier to conduct. Specifically, collecting samples of unrelated cases and controls is easier and less expensive than collecting family-based samples [1]. The goal of the case-control studies is to identify SNPs associated with the outcome of interest, such as disease status or responder/non-responder status.

GWA studies involve large amounts of data. For example, the Illumina Human1M Duo BeadChip has more than 1 million genetic markers per sample, and Affymetrix Genome-Wide Human SNP Array 6.0 features more than 1.8 million genetic markers. Proper statistical methods are needed to analyze such large datasets in order to draw meaningful conclusions. There are several steps involved in the analysis of the GWA case-control studies: 1) preprocess the raw data to give the genotype calls and filter out certain SNPs and samples based on quality control criteria [2,3]; 2) perform preliminary analyses, including computing allele and genotype frequencies, and testing Hardy-Weinberg equilibrium and linkage disequilibrium (See Balding at al. [4] and Gordon et al. [5] for an overview); 3) identify SNPs or haplotypes related to the outcome of interest while controlling false-positive findings by identifying and adjusting population stratification, performing association analysis using SNPs or haplotypes, etc. While there are challenging statistical issues implicated at each step, we will focus on the correction for population stratification involved in step 3.

In the population-based GWA case-control studies, it is assumed that the case and control participants are sampled from the same population, so the differences in allele frequencies are related only to the outcome of interest, instead of being related to background population differences between cases and controls. However, if this assumption is not met, it can cause spurious associations.

Population stratification (PS) refers to allele frequency differences between cases and controls unrelated to the outcome of interest, but due to sampling from populations with different ancestries. Correcting for population stratification is very important in GWA studies [6] since it can cause false positive findings. Large-scale GWA studies with many subjects are particularly vulnerable to population stratification artifacts [7,8]. Because of the large number of subjects, it is likely that there are some unrecognized hidden population structures that may be responsible for systematic differences being detected in SNPs between cases and controls.

A number of methods have been proposed to overcome confounding effects due to population stratification, and these have proven useful in certain situations. Two earlier approaches are the genomic control approach and the structured association approach. The genomic control (GC) approach [9] modifies the association test statistic by a common factor for all SNPs to correct for PS. This uniform correction can over-adjust or under-adjust certain SNPs, depending on the ancestral information of individual SNPs [10]. The structured association approach [11] tries to assign the samples to discrete subpopulation clusters and then accumulates evidence of association within each cluster. The structured association approach can be useful for small datasets (http://pritch.bsd.uchicago.edu/software/structure2_1.html). However, the STRUCTURE program can be computationally intensive and unwieldy for large scale GWA studies [10].

Currently, a widely used approach is the principal component analysis (PCA) approach, which was proposed by Price et al. [10]. In that paper, the EIGENSTRAT method based on PCA identifies several top principal components (PCs) and uses them as covariates in the association analyses. The PCA approach can be easily applied to thousands of markers, and the correction is specific to a marker’s variation in allele frequency across ancestral populations. This approach has been widely used in GWA studies [12,13], etc. However, the PCA approach may not adequately adjust for PS if the PS is due to the presence of several discrete subpopulations, since PCA uses the identified eigenvectors as continuous covariates. In addition, if there are outliers, the results based on the PCA adjustment may be misleading.

Li and Yu [8] proposed to combine multidimensional scaling (MDS) and clustering to deal with PS. Since MDS is equivalent to PCA for certain similarity matrices, that paper is essentially an extension of the PCA approach by adding subpopulation membership information. That approach can adjust for PS due to both discrete and continuous population structures, and it performs well for both large scale GWA studies as well as for smaller studies. However it has similar disadvantages to PCA as far as outliers are concerned, which will be shown in our simulation studies.

The linear mixed model has also been proven useful theoretically but it is computationally intensive. Recently, Zhang et al. [14] and Kang et al. [15] have proposed practically effective approaches to apply the linear mixed model to large scale GWAS studies to deal with population stratification and account for family structure and cryptic relatedness. Their methods have been implemented in software programs TASSEL and EMMAX respectively. However, the results based on these approaches are influenced by outliers as well.

In this paper, we propose to combine the clustering method used in Li and Yu [8] with robust PCA as an improved approach for correcting for artifacts arising from population stratification. The advantage of our approach is that it can deal with both discrete and continuous population structures, in the presence of subject outliers. Through simulation studies, we show that even a small percentage of outliers can greatly influence the analysis results from some widely used methods. This critical goal of handling the outliers properly is our motivation to propose new robust methods. We compared our proposed robust methods with several widely used methods using simulations and we demonstrated the usefulness of our proposed methods under various scenarios involving discrete and admixed population structures.

## Methods

### Summary of the procedure

We write the SNP data as an *n* by *p* matrix **X**, with rows representing the *n* subjects, and columns representing the *p* SNPs. The steps of the procedure are described below.

First, we identify subject outliers using a robust PCA approach based on the GRID algorithm [16] or the resampling by half means (RHM) approach [17]. Both approaches can handle the issue of large number of variables (*n*<*p*).

Second, we perform regular PCA on the SNP data matrix after removing the subject outliers, and select several top PCs. We apply the k-medoids clustering method [18] to the selected PCs, decide on the optimal number of clusters based on Gap statistics [19], and then assign each subject to a cluster.

Third, we test each SNP’s association with the outcome of interest by building a logistic regression model that includes the specific SNP as one factor, the selected PCs as covariates, and the cluster membership indicators as additional factors.

We explain the details of each step in the following sections.

### Robust PCA for outlier detection

There are a number of robust PCA approaches for multivariate data, such as minimum volume ellipsoid (MVE) [20], minimum covariance determinant (MCD) [21], as well as certain modifications of these methods. However, these approaches require the number of samples (subjects) to be larger than the number of variables (*n*>*p*). For example, the MCD estimator tries to identify a subset of size *h* for which the classical covariance matrix has a minimal determinant, and it is popular because of its high resistance to subject outliers. However, it cannot be used in studies involving a large number of variables (*n*<*p*) because the determinant of a covariance matrix of *n*<*p* observations will always be zero.

#### Projection pursuit robust PCA

The robust PCA based on the projection pursuit (PP) approach [16,22,23] can overcome the issue of a large number of variables (*n*<*p*). This approach does not use the covariance matrix, so it does not have the drawback of the covariance-based estimates that require that the number of samples be larger than the number of variables.

In the classical PCA the variances of the data on the projected directions are maximized. The robust PCA using the PP approach replaces the variance with a robust scale estimator *S*_*n*_ called the PP index. For example, *S*_*n*_ can be the median absolute deviation (MAD) [16,23]. For a sample {*z*_*1*_*,…,z*_*n*_}, MAD is defined as

$$\text{MAD}(z_{1},\ldots ,z_{n})=1.4826\underset{j}{\text{median}}|z_{j}-\underset{i}{\text{median}}(z_{i})|$$

If ***x***_***1***_***, …, x***_***n***_ denote the *n* rows (observations) of the data matrix **X**, the first principal component can be obtained by finding the vector **b** that maximizes the robust scale estimator *S*_*n*_ of the projected data:

$$b_{1}=\underset{||a||=1}{\text{argmax}S_{n}}(a^{t}x_{1},\ldots ,a^{t}x_{n}).$$

This method was first proposed by Li and Chen [22], who proved that this estimator is consistent, qualitatively robust, and inherits the breakdown point of the robust scale estimator. However, the algorithm they proposed is very complicated and difficult to apply in practice. Later improved algorithms have been proposed [16,23], etc. to make the method practical. The robust PCA based on the PP approach searches for eigenvectors sequentially. Thus in high dimensional SNP data, we only need to compute the top eigenvectors that we are interested in with reduced computational time.

In this paper, we considered two algorithms for the projection pursuit robust PCA: the CR algorithm proposed by Croux and Ruiz-Gazen [23], and the GRID algorithm proposed by Croux et al. [16].

Let *X* be a *n* (subjects) by *p* (variables) matrix, *x*_*i*_ be the vector for subject i, and $\hat{\mu }\left(X\right)$ be a location estimate vector for *X*, such as the median of X. Let K be the number of components that we want to compute and let *S*_*n*_ be the chosen robust scale estimator. The CR algorithm is as follows:

(i) To compute the first component (*k* =1), we first normalize the data by subtracting the centers of the variables $x_{i}^{1}=x_{i}-\hat{\text{μ}}\left(X\right)$ for *i* =1, 2, …, *n*. Define $A_{n,1}\left(X\right)=\left\{\frac{x_{i}^{1}}{\left|\left|x_{i}^{1}\right|\right|};1\;\leq \;i\;\leq \;n\right\},$ and the first eigenvector can be obtained as ${\hat{b}}_{1}=\underset{a\in \text{An},1\left(X\right)}{\text{argmax}S_{n}}\left(a^{t}x_{1}^{1},\ldots ,a^{t}x_{n}^{1}\right)$ and the first eigenvalue can be obtained by ${\hat{\lambda }}_{1}=S_{n}{}^{2}\left(\left({\hat{b}}_{1}^{t}x_{1}^{1},\ldots ,{\hat{b}}_{1}^{t}x_{n}^{1}\right)\right)$. Then the scores for the first component can be computed as $y_{i}^{1}={\hat{b}}_{1}^{\text{t}}x_{i}^{1}$ for *i*=1,…,*n*.

(ii)  To compute the *k*th component (*k*=2,…,*K*), define $x_{i}^{k}=x_{i}^{k-1}-y_{i}^{k-1}{\hat{b}}_{k-1}$ for *i*=1,…,*n*, $A_{n,k}\left(X\right)=\left\{\frac{x_{i}^{\text{k}}}{\left|\left|x_{i}^{k}\right|\right|};1\;\leq \;i\;\leq \;n\right\}$, the estimated eigenvector ${\hat{b}}_{k}=\underset{a\in A_{n,k}\left(X\right)}{\text{argmax}S_{n}}\;\left(a^{t}x_{1}^{k},\ldots ,a^{t}x_{n}^{k}\right)$, and the estimated scores $y_{i}^{k}={\hat{b}}_{k}^{t}x_{i}^{k}$ for the *k*th component.

The *k*th eigenvalue for *k* = 1 ,…, *K* is approximated by ${\hat{\lambda }}_{k}=S_{n}{}^{2}\left(\left({\hat{b}}_{k}^{t}x_{1}^{k},\ldots ,{\hat{b}}_{k}^{t}x_{n}^{k}\right)\right)$, and the robust covariance estimate can be calculated as $C_{\mathrm{Sn}}=\sum_{k=1}^{K}{\hat{\lambda }}_{k}{\hat{b}}_{k}{\hat{b}}_{k}^{t}$.

Croux et al. [16] proposed an improved algorithm called GRID. The basic idea of the GRID algorithm is to perform optimization using grid search. In the case of two dimensions (*p*=2), the optimization problem reduces to maximizing the function *θ*→*S*((cos(*θ*), sin(*θ*)))^*t*^ over the interval [−*π*/2,*π*/2], which can be done using a grid search. That is, we divide the interval into a number of equal-sized sub-intervals (for example, *J*-1 sub-intervals), and evaluate the function at the grid points $\left(-\frac{1}{2}+\frac{j}{J}\right)\pi$ for *j*=1,…,*J*. We can arrive at a good approximation to the solution if *J* is large enough. For the general case of *p*>2, we can perform iterative optimizations in two-dimensional space; for details, see Croux et al. [16].

In our simulations, we applied both the CR algorithm and the GRID algorithm. The CR algorithm tended to identify more observations as outliers compared to the GRID algorithm, but the results based on the CR algorithm and the GRID algorithm were similar in many cases of our simulations. Croux et al. [16] pointed out that the CR algorithm may have a swamping effect (meaning that good observations are incorrectly flagged as outliers) especially for small sample size with *p*>>*n*. As the number of variables *p* increases, the swamping effect may get worse. Some simulations with 100 subjects and 20,000 SNPs did show some swamping effect of the CR algorithm (data not shown). In a real GWA study, for example an Illumina 550 K chip, we can have 545,080 SNPs. After quality control and pruning based on the correlation between SNPs, we can still have several thousands to tens of thousands of SNPs that will be used for detecting outliers and adjusting for population structures. Thus for the GWA studies, the projection pursuit robust PCA based on the GRID algorithm is recommended, and the results based on the GRID algorithm were presented in this paper.

#### Outlier detection using robust PCA

Hubert et al. [24] proposed a diagnostic plot to identify different types of outliers. The plot is based on the score distance and the orthogonal distance of each observation. Denote the right robust eigenvector matrix corresponding to the variables as P_p_,_k_, and the robust location estimate (column vector) as $\hat{\mu }$. The robust score matrix is given as

Tn,k=Xn,p−1nμ^'Pp,k

The robust score matrix contains the robust scores of each subject (row) based on each of the first *k* components.

The score distance is given by

$$SD_{i}=\sqrt{t_{i}^{'}L^{-1}t_{i}}=\sqrt{\sum_{j=1}^{k}\left(t_{\mathrm{ij}}^{2}/l_{j}\right)}$$

where *t*_*ij*_ is an element of the robust score matrix and *l*_*j*_ is the jth eigenvalue, *i=1,…,n* (number of observation), and *j=1,…,k* (number of selected principal components). The cutoff value for the score distance is taken as square root of the 0.975^th^ quantile of *χ*_*k*_^2^ distribution, i.e.,

$$C_{\text{score}}=\sqrt{\chi_{k,0.975}^{2}}$$

The orthogonal distance measures the distance between an observation and its projection in the *k*-dimensional PCA subspace. It is defined as

$$OD_{i}=||x_{i}-{\hat{x}}_{i}||$$

where *x*_*i*_ is the *i*th vector (row) in the original data matrix X, and ${\hat{x}}_{i}$ is the estimated vector in the PCA subspace. To obtain the cutoff for the orthogonal distance, Hubert and Driessen [25] proposed to approximate the squared orthogonal distances by a scaled χ^2^ distribution with *g*_*1*_ degrees of freedom $OD^{2}\sim g_{2}\chi_{g_{1}}^{2}$ Robust estimates for *g*_*1*_ and *g*_*2*_ are derived using the Wilson-Hilferty transformation [26] to normality. Todorov and Filzmoser [27] have implemented a number of robust PCA methods, including a projection pursuit method, in an R package rrcov, which is available from Comprehensive R Archive Network (CRAN) at http://CRAN.R-project.org.

The score distance and orthogonal distance define four types of observations. The observations with small score distances and small orthogonal distances are the regular observations, and they form one homogeneous group that is close to the PCA subspace. The observations with large score distances and small orthogonal distances lie close to the space spanned by the PCA components, but far from the regular observations. This means that they are different from the regular observations, but there is not much loss of information when we use their fitted values in the PCA subspace. We call these observations type A outliers. The observations with large orthogonal distances but small score distances cannot be distinguished from the regular observations once projected onto the PCA subspace, but they lie far from this PCA subspace. This means that there is a considerable loss of information if we use their fitted values in the projected PCA subspace. We call these observations type B outliers. The observations with large score distances and large orthogonal distances lie far from the PCA subspace and after projection also far from the regular observations in the PCA subspace. We call these observations type C outliers. For the purpose of population stratification adjustment and association testing, we need to remove all the three types of outliers. The type C outliers will definitely need to be removed since they typically have a large influence on classical PCA as the eigenvectors will be shifted toward them. The type A outliers need to be removed since they are different from the regular observations and will influence the population stratification adjustment. And we also need to remove the type B outliers since they may influence the association tests. The type C and type A outliers will have a greater impact on the calculated eigenvectors used to adjust population stratification and thus a more pronounced impact on the GWA results compared with the type B outliers.

### Resampling by half means (RHM) for outlier detection

Resampling by half means (RHM) is another outlier detection approach for multivariate data that can overcome the issue of a large number of variables (*n*<*p).* This method was proposed by Egan and Morgan [17] and applied in chemometrics. It is an easy to understand method and we have implemented it in R. To start RHM, we can randomly select half of the total observations. The sampled data matrix is written as a *n*/2 by *p* matrix X_s_(i), and the mean m(i) and standard deviation s(i) vectors are determined. The original data matrix X is then scaled using m(i) and s(i) to arrive at a *n* by *p* scaled matrix X(i).

The Euclidean distance is calculated for each observation (row) and a *n* by 1 vector of lengths *l* (i) is obtained. All vector lengths are then stacked into a *n* by nrep (number of sampled data matrices) matrix **L**. We can then calculate the mean for each observation (row), and all the means form a n by 1 vector *xl*. A cutoff point c is defined to identify outlier observations. The plot of the mean vector lengths can be used to identify the outliers. In our application, those mean vector lengths that are bigger than the median+3*MAD are defined as outliers, where MAD is defined as $\text{MAD}=1.4826\underset{i}{\text{median}}\{|xl_{i}-\underset{j}{\text{median}}\left(xl_{j}\right)|\}$.

### Clustering based on principal components

After outlier detection using either robust PCA or RHM, classical PCA can be applied to the outlier-removed genotype data matrix. To decide on the number of components, we used the Tracy-Widom statistic [28] to test the number of significant eigenvalues, as in Price et al. [10]. The scree plot of the eigenvalues can also be used to decide upon the number of components. The cluster membership was obtained using the *k*-medoids clustering method [18], and the number of clusters was obtained using the Gap statistic [19] as in Li and Yu [8].

The *k*-medoids clustering method is more robust to outliers than the *k*-means clustering method. Compared with the *k*-means clustering method, the *k*-medoids clustering method requires the cluster center to be an observation instead of the calculated mean based on the observations and it minimizes a sum of pair-wise dissimilarities instead of a sum of squared Euclidean distances. Even though the outliers have been removed in the previous step based on robust PCA, it is still better to use a robust clustering method as a prudent step.

For a given number of clusters *k* ranging from 1 to *K*, the Gap statistic is defined as the log difference between the averaged within-cluster dispersions from the *B* sets of simulated datasets with no clusters and the within-cluster dispersion of the observed data.

The estimated number of clusters is the smallest k that satisfies Gap(*k*)≥Gap(*k*+1)-σ_*k*+1_, where σ_*k*+1_ is the standard deviation of the B replicates of log within-cluster dispersions from the simulated datasets. In our simulations, we set B=1000.

In cases when there are missing values, the alternating least squares approach [29,30] can be used to obtain the PCs. We start with an estimate of the first right eigenvector, and we regress each row of the original data matrix against the estimated first right eigenvector using a model with no-intercept. This gives a vector (*n* by 1) of coefficients. Now we regress each column of the original data matrix against this new coefficient vector with no-intercept and we obtain an updated (*p* by 1) estimate for the first right eigenvector. We keep alternating the regressions until we identify the first right eigenvector and the first left eigenvector. Then we can modify the original data matrix by subtracting the first principal component based on the first right and left eigenvectors, and generate the second set of left and right eigenvectors by applying alternating regressions on the modified data matrix with first principal component removed.

### Association testing using logistic regression models

To perform the association analysis for each SNP, a logistic regression model was used with the specific SNP as one factor, the PCs from the robust method as the covariates, and the cluster membership indicators as additional factors, as in Li and Yu [8]. The model is

$$\text{logit}\;\left(Y\right)=\beta g+\gamma X+\eta Z,$$

where Y represents the binary response variable (such as the disease status), *g* represents the genotype value of the specific SNP, X represents the PCs from the robust method, and Z represents the cluster membership indicators. In this model, the principal components adjust for the continuous population structure and the class membership indicators adjust for the discrete population structure. To test whether there is an association between the specific SNP *g* and the binary response Y, a likelihood ratio test can be used to compare the model with and without SNP *g*, or a Wald test can be used to test the statistical significance of SNP *g* adjusted for covariates X and Z. If multiple SNPs are tested, multiplicity adjustment methods, such as Bonferroni-Holm method [31] or Benjamin-Hochberg false discovery rate method [32], can be used.

### Simulations

Simulations were used to compare six different methods: the likelihood ratio test (LRT) without PS adjustment (Trend) [8], the genomic control method (GC) [9], the PCA method [10], the MDS method [8], the robust method using RHM and PCA (RPCA-RHM), and the robust method using PP robust PCA (RPCA-PP). We compared these methods with respect to their empirical false positive rate and true positive rate. The nominal level was set as 0.01. The empirical false positive rate was calculated based on situations when there were no associations between SNPs and the endpoint; while the true positive rate was calculated based on situations when there were associations between SNPs and the endpoint. We used simulated datasets with and without subject outliers. In simulations I and III, there were no outliers, while in simulations II and IV, subject outliers were added to the data.

#### Design for simulation I

As in Price et al. [10], for each subpopulation, the allele frequency for each SNP was generated independently from a beta distribution with two parameters, *p*(1-F_ST_)/F_ST_, (1-*p*)(1-F_ST_)/F_ST_, where the inbreeding coefficient F_ST_ was set to 0.01 (F_ST_ of 0.01 is typical of differentiation between divergent European populations) and the ancestral population allele frequency *p* was simulated from the uniform distribution on [0.1,0.9]. Assuming two or three underlying populations, we simulated 500 cases and 500 controls. We used the genotypes of 2000 disease-unrelated SNPs to correct for PS. The details for each scenario are shown in Table 1, where there are two underlying populations in S1 and S2, and there are three underlying populations in S3 and S4. These scenarios were the same as those used in Li and Yu [8] to perform method comparisons.

**Table 1:** Population stratification configurations in simulations I and II

		**Case**	**Control**
		**proportion**	**proportion**
S1	(moderate)	(0.6,0.4)^a^	(0.4,0.6)^b^
S2	(more extreme)	(0.5,0.5)	(0,1)
S3	(moderate)	(0.45,0.35,0.20)	(0.35,0.20,0.45)
S4	(more extreme)	(0.33,0.67,0)	(0,0.33,0.67)

^a^ The proportion of cases sampled from each subpopulation.

^b^ The proportion of controls sampled from each subpopulation.

To evaluate the performance of the different methods in association testing, we simulated three types of testing SNPs and applied the different methods to test the association between the testing SNP and the binary endpoint (case or control). The first type included the random SNPs with no association to the disease. These SNPs were generated the same way as those SNPs chosen for detecting the population stratification. The second type included the differential SNPs with no association to the disease. These SNPs have high allele frequencies differences between subpopulations. In our simulations, the allele frequency for population 1 was 0.8, while the allele frequency for population 2 was 0.2. The third type included the causal SNPs that were associated with the disease. We assume a relative risk of *R*=1.3 for the causal allele similar to Li and Yu [8]. The risk model with a relative risk R for the causal allele was generated as follows: for individuals from population *l* with population allele frequency *p*_*l*_, control individuals were assigned genotype 0, 1, or 2 with probabilities *(1 – p*_*l*_*)*^*2*^, *2p*_*l*_*(1 – p*_*l*_), or *p*^*2*^_*l*_, respectively, and case individuals were assigned genotype 0, 1, or 2 with relative probabilities *(1 – p*_*l*_*)*^*2*^, *2p*_*l*_*(1 – p*_*l*_*)*, or *p*^*2*^_*l*_, respectively, and case individuals were assigned genotype 0, 1, or 2 with relative probabilities *(1 – p*_*l*_*)*^*2*^, *2Rp*_*l*_*(1 – p*_*l*_*)*, or *R*^*2*^*p*^*2*^_*l*_, respectively, each scaled by *(1 – p*_*l*_*)*^*2*^*+ 2Rp*_*l*_*(1 – p*_*l*_*) + R*^*2*^*p*^*2*^_*l*_.

To evaluate the false positive rate and true positive rate, we generated 100 datasets including 500 cases and 500 controls. Each dataset contained 2000 disease-unrelated SNPs which were used to adjust PS and 1000 testing SNPs for each category (random, differential, or causal). The same numbers of testing SNPs were used in Li and Yu [7].

#### Design for simulation II

Simulation II data were generated by adding subject outliers to the simulation I data. Five percent outlier subjects were generated by replacing 5% of the 2nd eigenvector values corresponding to the subjects with extreme values, and then reconstructing the SNP data matrices. The detailed steps are as follows. First, generate the simulated data as in simulation I. Second, apply singular value decomposition to the simulated data X and obtain the left eigenvectors corresponding to the subjects (U), right eigenvectors corresponding to the SNPs (V) and eigenvalues (d), where X=UdV^T^. For example, the second left eigenvector contained 1000 values and corresponded to the 1000 subjects. Third, replace 5% of the values in the second left eigenvector with extreme values, and call the modified left eigenvectors U_mod_. Fourth, reconstruct the data matrix back using the modified second left eigenvector together with the other eigenvectors and eigenvalues from the originally simulated data matrix. That is, X_mod_= U_mod_dV^T^. Fifth, since we are generating SNP data, replace all those values smaller than 0 in X_mod_ with 0, and all those values greater than 2 with 2. This will give us a modified data matrix with 5% subject outliers.

To evaluate the false positive rate and true positive rate, we generated 100 datasets including 500 cases and 500 controls. Each dataset contained 2000 disease-unrelated SNPs which were used to adjust PS and 1000 testing SNPs for each category (random, differential, or causal).

#### Design for simulations III and IV

In simulation III, we generated an admixed population with two ancestral populations. As in Price et al. [9], the disease status for individuals with proportions *a* from population 1 and (1-*a*) from population 2 was simulated using disease risk proportional to *r*^*a*^, where *a* is uniformly distributed on (0,1) and *r* is the ancestry risk, set to 3 in our simulations. To obtain an average value of 0.5 across all possible values of *a*, the probability of disease was set to 0.5log(*r*)*r*^*a*^/(*r*−1). The risk model with a relative risk of *R*=1.3 for the causal allele was implemented as in the discrete cases, by replacing *p*_*l*_ with *ap*_*1*_*+ (1 – a)p*_*2*_, the allele frequency conditional on an individual’s ancestry proportion *a.*

Simulation IV data were generated by adding subject outliers to the simulation III data. As described previously, 5% outlier samples were generated by replacing 5% of the 2nd eigenvector values with extreme values and then reconstructing the SNP data matrices.

For both simulations III and IV, we generated 20 datasets of 500 cases and 500 controls. Each dataset contained 20,000 substructure inference SNPs and 1000 testing SNPs for each category (random, differential, or causal). Compared to simulations I and II, we have more substructure inference SNPs since more SNPs are needed to identify the population structures in the admixed populations than in the discrete populations.

## Results and discussion

For each population stratification simulation scenario, the empirical false positive rate and true positive rate were estimated by averaging the results corresponding to the 1000 SNPs from each category of the simulated datasets. The nominal significance level was chosen to be 0.01.

### Simulation I results

The results for simulation I are listed in Table 2. In simulation I, there were no outliers. As can be seen, the empirical false positive rates for the Trend method were inflated for both random and differentiated SNPs. Using the GC method, the false positive rates for random SNPs were less than or close to the nominal level, but the false positive rates for differentiated SNPs were inflated considerably. As for the PCA method, when there were moderate differences between cases and controls, the false positive rates for random SNPs and differentiated SNPs were close to the nominal level; for more extreme differences between cases and controls, the false positive rates for random SNPs were under control, but the false positive rates for differentiated SNPs were inflated. As expected, in the absence of outliers, the performance of the MDS, RPCA-RHM, and RPCA-PP methods was similar. The empirical false positive rates for random SNPs and differentiated SNPs were close to the nominal level.

**Table 2:** Empirical false positive rate and true positive rate results for simulation I (Discrete Populations without Outliers)

**Case control**	**Testing SNP**	**Trend**	**GC**	**PCA**	**MDS**	**RPCA-**	**RPCA-**
**Difference**	**Types**					**RHM**	**PP**
S1	Random SNPs	2.67	0.91	0.97	0.97	0.99	0.97
(2 populations, moderate)	Differentiated SNPs	99.85	98.86	1.30	0.90	0.88	0.89
	Causal SNPs	48.99	34.13	47.37	47.29	46.92	47.33
S2	Random SNPs	16.56	0.89	1.11	0.92	0.93	0.92
(2 populations, more extreme)	Differentiated SNPs	100.00	100.00	13.60	1.00	1.01	0.99
	Causal SNPs	49.91	10.91	33.89	31.76	31.63	31.77
S3	Random SNPs	3.14	0.97	0.94	0.93	0.95	0.92
(3 populations, moderate)	Differentiated SNPs	99.99	99.98	2.24	1.00	1.01	1.00
	Causal SNPs	48.18	31.76	45.16	45.08	44.60	45.09
S4	Random SNPs	21.76	0.94	1.45	1.05	1.05	1.06
(3 populations, more extreme)	Differentiated SNPs	100.00	100.00	41.78	0.96	0.95	0.96
	Causal SNPs	50.79	8.42	23.51	19.34	19.13	19.34

^a^For random SNPs and differentiated SNPs, the values in the table represent the empirical false positive rates; for causal SNPs, the values in the table represent the empirical true positive rates. The nominal false positive rate is 0.01. Note that the numbers in the table refer to percentages.

### Simulation II results

**Figure 1:**
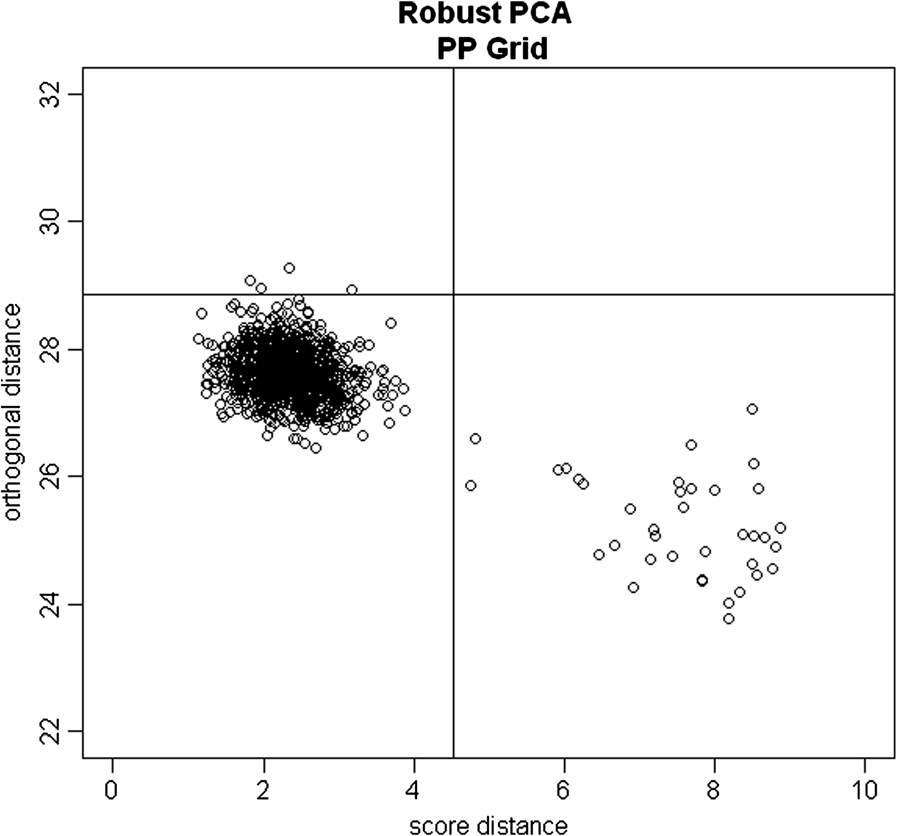
**The orthogonal distance versus the score distance for one simulated dataset.** The plot is based on projection pursuit robust PCA using the GRID algorithm for one simulated dataset under scenario S4 in simulation II. The vertical line is the outlier cutoff line for the score distance, the horizontal line is the outlier cutoff for the orthogonal distance, and those points on the right of the vertical line or above the horizontal line were identified as outliers.

Simulation II data were generated by adding outliers to the simulation I data. The results, summarized in Table 3, reveal that when using the Trend method, the empirical false positive rates for random SNPs were somewhat inflated while the empirical false positive rates for differentiated SNPs were substantially inflated. Using the GC approach, the false positive rates for random SNPs were modestly inflated, while the false positive rates for differentiated SNPs were substantially inflated. Using the PCA method, the false positive rates for random SNPs were somewhat inflated, while the false positive rate s for differentiated SNPs were considerably inflated. The MDS approach performed well under the scenarios of moderate case control differences, but the false positive rates for differentiated SNPs were moderately inflated under the scenarios of more extreme case control differences. Both the RPCA-RHM and RPCA-PP methods performed well, and the false positive rates for random SNPs and for differentiated SNPs were close to the nominal levels. The empirical true positive rates of the RPCA-RHM method and RPCA-PP method were comparable. Figure 1 shows the plot of orthogonal distances versus score distances for one simulated dataset under scenario S4. The majority of the data points cluster on the lower left corner, while the subject outliers are scattered on the right side of the vertical line or above the horizontal line.

**Table 3:** Empirical false positive rate and true positive rate results for simulation II (Discrete Populations with Outliers)

**Case control**	**Testing SNP**	**Trend**	**GC**	**PCA**	**MDS**	**RPCA-**	**RPCA-**
**Difference**	**Types**					**RHM**	**PP**
S1	Random SNPs	2.75	1.41	1.94	0.97	1.01	0.99
(2 populations, moderate)	Differentiated SNPs	99.85	98.75	93.03	1.33	0.99	1.00
	Causal SNPs	48.97	37.55	48.33	46.95	44.69	45.06
S2	Random SNPs	16.74	1.71	8.38	1.09	0.99	1.00
(2 populations, more extreme)	Differentiated SNPs	100.00	100.00	100.00	6.91	1.14	1.29
	Causal SNPs	49.94	14.09	44.77	32.81	30.07	30.21
				
S3	Random SNPs	3.40	1.12	1.65	1.08	1.06	1.06
(3 populations, moderate)	Differentiated SNPs	100.00	99.99	63.28	1.36	1.02	1.02
	Causal SNPs	48.85	31.61	46.72	45.81	43.29	43.89
						
S4	Random SNPs	21.35	1.15	9.82	1.10	0.92	0.97
(3 populations, more extreme)	Differentiated SNPs	100.00	100.00	100.00	18.13	1.29	1.51
	Causal SNPs	50.09	9.41	37.56	21.76	18.66	18.81

^a^For random SNPs and differentiated SNPs, the values in the table represent the empirical false positive rates; for causal SNPs, the values in the table represent the empirical true positive rates. The nominal false positive rate is 0.01. Note that the numbers in the table refer to percentages.

### Simulations III and IV results

Table 4 shows the results for the admixed populations from simulations III and IV. As we can see, using the Trend test, the empirical false positive rates for random SNPs were modestly inflated, but the false positive rates for differentiated SNPs were more substantially inflated. For the GC method, the false positive rates for random SNPs were close to the nominal level, but the false positive rates for differentiated SNPs were inflated quite substantially. For the PCA and MDS methods, the false positive rates for random SNPs and differentiated SNPs were close to the nominal level if there were no outliers; however, the false positive rates for differentiated SNPs were highly inflated if there were outliers in the data. Both the RPCA-RHM and RPCA-PP methods performed well, and the false positive rates for random SNPs and for differentiated SNPs were close to the nominal levels. The empirical true positive rate of the RPCA-RHM and RPCA-PP methods were comparable.

**Table 4:** Empirical false positive rate and true positive rate results for simulations III and IV (Admixed populations)

**Case**	**Testing SNP**	**Trend**	**GC**	**PCA**	**MDS**	**RPCA-**	**RPCA-**
**Control**	**Types**					**RHM**	**PP**
**Difference**							
Simulation III	Random SNPs	2.09	0.91	0.90	0.89	0.91	1.10
(no outliers)	Differentiated SNPs	97.16	94.29	1.12	1.09	1.09	1.10
	Causal SNPs	49.22	36.88	45.09	45.06	44.64	44.10
Simulation IV	Random SNPs	2.27	1.12	1.89	1.04	0.91	0.80
(with outliers)	Differentiated SNPs	97.59	94.17	88.11	10.09	1.01	1.40
	Causal SNPs	49.15	37.63	48.23	45.30	42.37	45.50

^a^For random SNPs and differentiated SNPs, the values in the table represent the empirical false positive rates; for causal SNPs, the values in the table represent the empirical true positive rates. The nominal false positive rate is 0.01. Note that the numbers in the table refer to percentages.

### Application to rheumatoid arthritis study

We applied our proposed method to a rheumatoid arthritis (RA) GWAS data used in a genetic analysis workshops (GAW16). This dataset, provided by the North American Rheumatoid Arthritis Consortium (NARAC), involved 868 RA cases and 1194 controls. There were 545,080 SNPs available for analysis.

Quality control of genotype data was conducted using PLINK as follows [33]. At the subject level, a call rate of at least 0.95 was required. At the SNP level, a call rate of at least 0.95, a minor allele frequency of at least 0.01, and a p-value of at least 10^-5^ from the Hardy-Weinberg equilibrium test were required. After the quality control step, we have 490,209 SNPs.

To perform population stratification, the remaining SNPs were further reduced as follows: (i) certain known high linkage disequilibrium (LD) regions were excluded (chr8:8000000..12000000, chr6:25000000..33500000, chr11:45000000..57000000, chr5:44000000..51500000); (ii) SNPs were pruned such that all SNPs within a window size of 1,500 (step size of 150) had pairwise r^2^<0.05; (iii) only autosomal SNPs were used. After pruning and filtering, 32,292 autosomal SNPs were kept. These SNPs were used to adjust population stratification using different methods.

**Figure 2:**
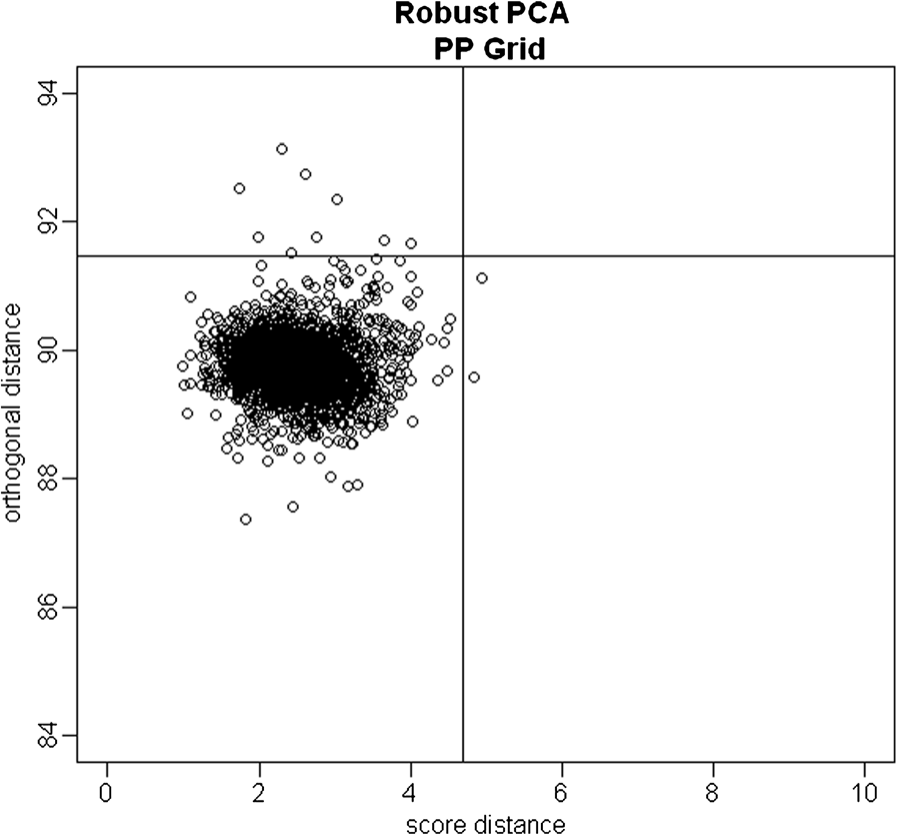
**The orthogonal distance versus the score distance for NARAC data.** The vertical line is the outlier cutoff line for the score distance, the horizontal line is the outlier cutoff for the orthogonal distance, and those points on the right of the vertical line or above the horizontal line were identified as outliers.

In the proposed robust methods, we first need to identify outliers. To do this, the 32,292 autosomal SNPs were further reduced by requiring that all SNPs within a window size of 1,500 had pairwise r^2^<0.02. This gave us 17,792 SNPs. The PP robust PCA was then applied on these reduced autosomal SNP sets. Figure 2 presents the diagnostic plot for outlier detection based on PP robust PCA using the Grid algorithm. Eleven subjects were identified as possible outliers. Among the 11 outliers, 2 subjects have large score distances and 9 have large orthogonal distances. We also applied the RHM method to identify outlier subjects. Among the eleven outliers identified by PP robust PCA, 7 subjects were also identified by RHM, and the other four were close to the cutoff of the RHM method. Since the PP robust PCA and RHM methods were consistent for this dataset, we used PP robust PCA method for further comparisons with several other existing methods.

**Figure 3:**
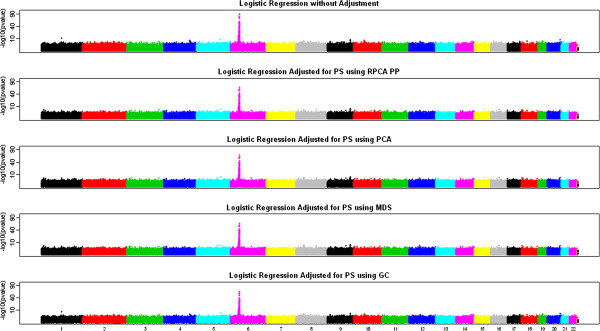
**Results of GWA analyses based on five different methods.** The y axis is in square root scale to improve readability.

To study the performance of different methods for the real dataset, we carried out association tests for all the SNPs (490,209 SNPs) using different methods. To adjust population stratification using PCA, MDS or Robust PCA, 32292 autosomal SNPs were used. For this dataset, we definitely need to take population stratification into consideration as the inflation factor is 1.43 without any adjustment. The PCA, MDS and Robust PCA methods were all able to adjust population structures and reduced the inflation factor to about 1.05. Figure 3 shows the results from the five GWA analyses using logistic regression without any adjustment, GC method, PCA method, MDS method and our proposed robust method using PP robust PCA. As we can see, all the methods were able to identify the HLA region on chromosome 6, which had been implicated in numerous rheumatoid arthritis (RA) studies [34-37]. Among the SNPs in the non-HLA region, the top three SNPs identified by robust PCA are on chromosome 9, a region that links to TRAF1, C5 and PHF19. TRAF1, C5 and PHF19 were reported to be associated with risk of RA in several studies [38-41]. As shown in Table 5 with both pvalues and rankings, these three SNPs were ranked at the top by three methods: robust PCA, PCA and MDS. However, robust PCA generated the most significant p-values. On the other hand, three SNPs (rs12913832, rs3930739, rs11632017) on chromosome 15 were found possibly associated with risk of RA by GC and Trend methods with p-values less than 0.0005, but not by robust PCA (p-values > 0.1), PCA (p-values > 0.05) or MDS (p-values > 0.05) at all. Further interrogation suggests that rs12913832 links to HERC2, and has been reported to be associated with hair colors; rs3930739 links to OCA2; and rs11632017 links to GABRG3. However, none of those three genes were reported to be associated with risk of RA. In this example, the GWA analysis results based on PCA, MDS and robust PCA were not dramatically different since there were no extreme outliers (outliers with very large score distances based on the diagnostic plot).

**Table 5:** Comparison of the analysis results for three SNPs on chromosome 9 known to be associated with RA

	**SNP rs2900180**		**SNP rs1953126**		**SNP rs881375**	
		**Rank in**		**Rank in**		**Rank in**
		**non-HLA**		**non-HLA**		**non-HLA**
	**p-Value**	**SNPs**	**p-Value**	**SNPs**	**p-Value**	**SNPs**
rPCA	1.15E-07	1	2.78E-07	2	3.20E-07	3
PCA	1.91E-07	1	4.71E-07	2	5.55E-07	3
MDS	1.69E-07	1	4.55E-07	2	4.91E-07	3
Trend	8.05E-09	4	3.52E-08	7	2.82E-08	6
GC	1.46E-06	4	4.13E-06	7	3.54E-06	6

## Conclusions

In GWA studies, properly adjusting for population stratification is extremely important. There are existing methods, such as the PCA and MDS methods, which have been proven to be highly useful for such large-scale studies. However, these methods are sensitive to outliers and may yield misleading results if there are outliers in the data. As it can be seen from our simulation studies, the false positive rates can be greatly inflated under certain scenarios if the outliers are not handled properly. One may argue that the classical PCA can also be used to identify outliers. However, we may not be able to identify all the outliers using the first few components from the classical PCA, and in fact artificial datasets can be constructed where all outliers remain masked by a classical PCA [23].

We herein propose robust methods for handling outliers and minimizing the confounding effects of population stratification in GWA studies. Our proposed methods can be considered as an extension of PCA and MDS methods to deal with outliers. We compared the performances of our proposed methods with several existing methods using simulation studies. For the two robust methods we proposed (RPCA-RHM and RPCA-PP), the false positive rates for random SNPs and differentiated SNPs were close to the nominal level in all the scenarios considered. Of the two robust methods proposed, both of them performed well in our simulations. The RPCA-PP method uses projection pursuit robust PCA to handle outliers, and a freely available R package can be used to perform projection pursuit robust PCA. The RPCA-RHM method uses a resampling by half means approach to handle outliers, and is quite straightforward in concept and easy to implement. However, RPCA-RHM may take longer for large datasets.

Overall, if there were no outliers in the data, our proposed methods were comparable to the best performing available methods. Importantly, if there were subject outliers in the data, our proposed methods performed superior to the other methods, especially for admixed populations and discrete populations with more extreme differences between cases and controls (S2 and S4 in Table 3, and Simulation IV in Table 4).

In this paper, we propose effective method to adjust for population structures. For well designed studies with unrelated subjects, embedded population structures may be the major concern. However, if some other sample structures such as family structures or cryptic relatedness are of concern, the linear mixed models [14,15,42] can be used. However, the results based on linear mixed model approaches are influenced by outliers based on our simulations (results not shown). In this case, our proposed methods can be extended to the linear mixed model setting to minimize confounding effects of population structures as well as family structures or cryptic relatedness.

To summarize, we demonstrate that subject outliers can greatly influence the analysis results in GWA studies. Our proposed robust methods outperform the existing population stratification methods in the presence of subject outliers. In practice, it is recommended to use robust population stratification methods in the analysis of GWA study data to avoid making inappropriate conclusions due to outliers.

## Competing interests

The authors declare that they have no competing interests.

## Authors’ contributions

LL proposed the methodologies and drafted the manuscript. LL and HL carried out the simulations. LL, DZ, HL, CA discussed the results and wrote the manuscript. All authors read and approved the final manuscript.
